# Improving Lunar Soil Simulant for Plant Cultivation: Earthworm-Mediated Organic Waste Integration and Plant-Microbe Interactions

**DOI:** 10.3390/plants14071046

**Published:** 2025-03-27

**Authors:** Zhongfu Wang, Sihan Hou, Boyang Liao, Zhikai Yao, Yuting Zhu, Hong Liu, Jiajie Feng

**Affiliations:** 1 Institute of Environmental Biology and Life Support Technology, School of Biological Science and Medical Engineering, Beihang University, Beijing 100191, China; wangzhongfu@buaa.edu.cn (Z.W.); by2310117@buaa.edu.cn (S.H.); liaoboyang@buaa.edu.cn (B.L.); yaozhikai@buaa.edu.cn (Z.Y.); zhuyut@buaa.edu.cn (Y.Z.); 2 International Joint Research Center of Aerospace Biotechnology & Medical Engineering, Beihang University, Beijing 100191, China; 3 Innovation Center for Medical Engineering & Engineering Medicine, Hangzhou International Innovation Institute, Beihang University, Hangzhou 311115, China

**Keywords:** lunar agriculture, Bioregenerative Life Support System (BLSS), earthworm, lunar soil cultivability

## Abstract

Long-term human residence on the Moon is an inevitable trend in lunar exploration, necessitating the development of Bioregenerative Life Support Systems (BLSSs). In BLSSs, plant cultivation serves as the core functional unit, requiring substantial amounts of cultivation substrates. Lunar soil has potential as a cultivation substrate, but its suitability for plant growth must be improved to meet life-support requirements. As a fine-grained, organics-free, in situ resource, lunar soil’s high compaction significantly restricts crops’ root access to oxygen, water, and nutrients. While the addition of organic solid waste—a byproduct of BLSSs—could alleviate compaction, issues such as salinization, incomplete decomposition, and the presence of pathogens pose risks to crop health. In this study, we introduced earthworms into wheat cultivation systems to gradually digest, transfer (as vermicompost), and mix solid waste with a lunar soil simulant substrate. We set five experimental groups: a positive control group using vermiculite (named as V) as the optimal growth substrate, a negative control group using pure lunar soil simulant (LS), and three treatment groups using lunar soil simulant with solid waste and 15 (LS+15ew), 30 (LS+30ew), and 45 (LS+45ew) earthworms added. Our results demonstrated significant improvements in both compaction (e.g., bulk density, hydraulic conductivity) and salinization (e.g., salinity, electrical conductivity), likely due to the improved soil aggregate structures, which increased the porosity and ion adsorption capacity of the soil. Additionally, the microbial community within the substrate shifted toward a cooperative pattern dominated by significantly enriched plant probiotics. Consequently, the cultivated wheat achieved approximately 80% of the growth parameters (including production) compared to the control group grown in vermiculite with nutrient solution (representing ideal cultivation conditions), indicating sufficient nutrient supply from the mineralized waste. We can conclude that the earthworms “complementarily” improved the lunar soil simulant and organic waste by addressing compaction and salinization, respectively, leading to comprehensive improvements in key parameters, including the microbial environment. This study proposes a conceptual framework for improving lunar soil for crop cultivation, and it innovatively introduces earthworms as a preliminary yet effective solution. These findings provide a feasible and inspiring foundation for future lunar agriculture.

## 1. Introduction

Space exploration is a fateful course for humanity’s future, and it is also a considerable stimulus for scientific research and the economy. As the Earth’s satellite, the Moon serves as the prime location for exploration. Crewed bases are the undoubted trend of lunar exploration, and to ensure long-term human residence on the Moon, Bioregenerative Life Support Systems (BLSSs) are imperative. BLSSs are closed artificial ecosystems providing essential life-supporting materials and comfortable environments similar to the Earth’s ecosystems for human survival [1]. As the core functional unit of BLSSs, the cultivation of higher plants can absorb CO_2_ and provide O_2_, clean water, and fresh food [2]. Plants also have a delightful ability to help maintain psychological health within closed environments [3]. Large-scale plant cultivation for long-term BLSSs requires a large amount of cultivation substrate. However, the long Earth–Moon distance adds to the difficulties and costs, thus complicating the cultivation substrate’s transportation from the Earth. Fortunately, lunar soil (also known as lunar regolith, or lunar dust for smaller particles), an organics-free, organism-free, and very fine-grained (~50 µm) mineral on the lunar surface, is an abundant in situ resource with considerable potential as a cultivation substrate. It is rich in plant mineral nutrient elements such as Si, Fe, P, and Ca [4]. Proper utilization of lunar soil as a plant cultivation substrate will considerably reduce the cost and increase the robustness of BLSSs, thereby improving the feasibility of crewed lunar exploration.

Many researchers have examined lunar soil’s feasibility as a plant cultivation substrate. Initially, lunar soil collected from the Apollo 11 and Apollo 12 missions was sprinkled on seeds, seedlings, and tissue cultures, and it was even rubbed on plant leaves, with the plant samples growing normally without phenotypic changes detected, indicating no toxicity to plants [5]. However, treating tobacco cells with Apollo 12 lunar soil resulted in increased chlorophyll and sterol levels, suggesting an impacted plant metabolism [6]. Tissue cultures of slash pine treated with Apollo 15 lunar soil decreased in fatty acid content, sterol content, and cell respiration, while they increased in cytoplasmic density [7]. These studies indicate no toxicity but certain effects of lunar soil on plant tissues. Most recently, in 2022, a real-sense “lunar-soil-cultivation” experiment was carried out. *Arabidopsis thaliana* was cultivated in 12 g of Apollo 11, Apollo 12, and Apollo 17 lunar soil in total, and the plant’s transcriptome was analyzed. The results showed that *Arabidopsis* was able to grow in lunar soil, but it exhibited slower growth and severely stressed phenotypes: genes related to ion/metal/reactive oxygen species (ROS) stresses were significantly upregulated [8]. Our closer observation revealed that the plant stress in lunar soil simulant—physically and chemically similar to real lunar soil—likely stems primarily from its fine-grained compaction, which leads to low porosity and, thus, suppressed access of the roots to oxygen, water, and nutrients [9]. Therefore, lunar soil is able to cultivate plants, but it needs to be improved to achieve better plant growth for the life-support needs of BLSSs.

Organic solid waste, which is incompletely fermented from plant residues and human feces by microbes, is a daily and inherent product of BLSSs [2]. In lunar surface BLSSs, solid waste also has potential as a cultivation substrate in situ [9]. Rich in organics and microbes, solid waste is able to mitigate the compaction and offer plant nutrients, but it is also too rich in salts (originally coming from plant nutrient solutions) and semi-decomposed organics, and thus also stressful to crops [9]. In contrast, lunar soil is too compact and lacks nutrients, yet it exhibits relatively low salt stress on crops and could thus serve as a buffering agent. That is, the advantages and disadvantages of the two potential substrates are potentially complementary to each other, and we are hence inspired to explore the possibility of inducing a synergistic improvement between them. In Earth’s ecosystems, earthworms are well-known as efficient “soil engineers” due to their capacity to increase aggregate structures [10], promote plant nutrient absorption through vermicomposting [11], and facilitate the colonization of plant probiotics [10]. The soil aggregate structures formed by earthworms would mitigate compaction and adsorb salts, thereby achieving complementarity and improving the cultivability. Practically, a recent study proved that *Eisenia fetida*, the most widely used earthworm genus, is able to survive in lunar soil simulant for 14 to 60 days [12]. Therefore, we anticipate promising prospects for incorporating earthworms into the cultivation substrate improvement systems of BLSSs.

In our previous study (without earthworms), leading to the current one, simply fermenting a mixture of lunar soil simulant and organic solid waste already significantly mitigated the substrate’s compaction, and correspondingly improved the cultivated wheat seedling lengths (reaching 64% of the vermiculite-cultivated), likely due to the effective microbial erosion of lunar soil simulant particles [9]. However, this production level needs to be further improved to meet the life-support requirements of BLSSs. Here, we conducted a whole-life-span wheat cultivation experiment (“from seed to seed”) using lunar soil simulant, and introducing earthworms to gradually digest, transfer, and mix the solid waste (at the diagonal-corner feeding zones) with the lunar soil simulant substrate. We performed a comprehensive analysis of the cultivation substrate, including its physical and chemical properties, the composition and interaction patterns of the microbial community, and the morphological, agronomic, and physiological parameters of wheat. This study advances our research series on lunar soil improvement as a cultivation substrate for space food by emphasizing the “symmetry” between lunar soil (compaction) and solid waste (salinization) and introducing (and validating) earthworms as effective catalysts of their complementary improvement process.

## 2. Results

### 2.1. Earthworms Mitigated the Substrate’s Salinization and Compaction

**Figure 1:**
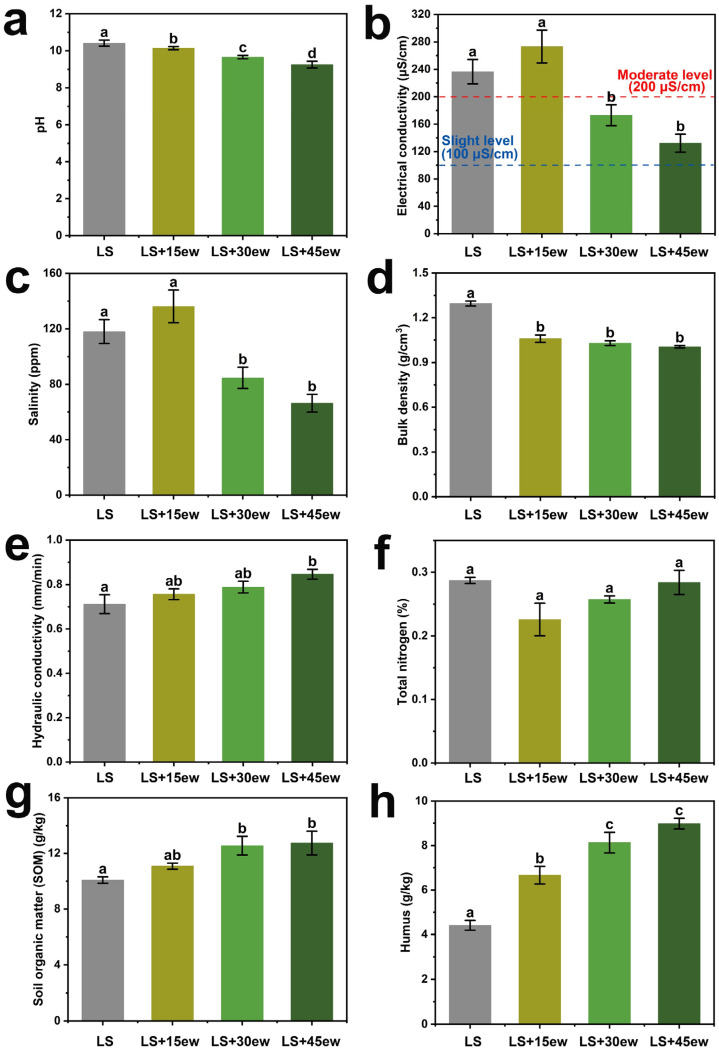
Environmental factors of lunar-soil-simulant-based cultivation substrates. (**a**–**h**) Substrate physical and chemical factors after the wheat cultivation. Data are means ± standard error (*n* = 5 independent replicates). Letters (i.e., a, b, c, and d) above the bars indicate the results of ANOVA’s post hoc LSD tests identifying significant differences (*p* < 0.05).

We measured the physical and chemical properties of the cultivation substrate after the wheat harvest (day 65), including salinization indicators (pH, electrical conductivity, and salinity), compaction indicators (hydraulic conductivity and bulk density), and the chemical composition indicators (total nitrogen, soil organic matter content (SOM), and humus content) (Figure 1a–h). Notably, SOM and humus also serve as indirect indicators of substrate compaction.

Likely due to the plant nutrient solution added, the pure lunar soil simulant (the earthworm- and solid-waste-free control) group exhibited significant salinization, with a pH of 10.42 (Figure 1a), an electrical conductivity of 236.58 μS/cm (Figure 1b), and a salinity of 118 ppm (Figure 1c), reaching the moderate level of salt-affected land [13]. Unsurprisingly, the pure lunar soil simulant also exhibited severe compaction, with the highest bulk density (Figure 1d) and the lowest hydraulic conductivity (Figure 1e) and humus content (Figure 1h).

With the addition of earthworms and solid waste (and no plant nutrient solution), generally, the substrate’s physical and chemical indicators showed successive improvement along the earthworm abundance gradient (Figure 1), manifested in the mitigation of both salinization and compaction. Earthworms alleviated the substrate’s compaction (likely caused by the lunar soil simulant) by significantly reducing bulk density (by 22.4% in the 45-earthworm treatment, Figure 1d) and increasing hydraulic conductivity (by 14%, Figure 1e), soil organic matter (SOM) content (by 24.9%, Figure 1g), and humus content (by 50.2%, Figure 1h). These improvements can likely be attributed to the organic waste transported from the feeding zone, potentially in the form of vermicompost (earthworm feces).

Regarding salinization, adding low-abundance earthworms (the 15 ew group) resulted in even higher ionic stress than the pure lunar soil simulant group (Figure 1b,c), possibly due to the transfer of the highly saline waste by earthworms to the wheat root zone. However, adding 30 or 45 earthworms largely mitigated the substrate’s salinization to an even lower level than in the lunar soil simulant group, including the electrical conductivity (Figure 1b) and salinity (Figure 1c) factors; this is probably due to the increased aggregate structure’s physical adsorption (Figure S1) and the better absorption/utilization in the grown wheat. Notably, the pH also showed a significant, successive neutralization along the earthworm abundance gradient (Figure 1a), which is a common benefit provided by earthworm amendments.

### 2.2. Substrate Microbial Communities Improved as Probiotic and Cooperative

**Figure 2:**
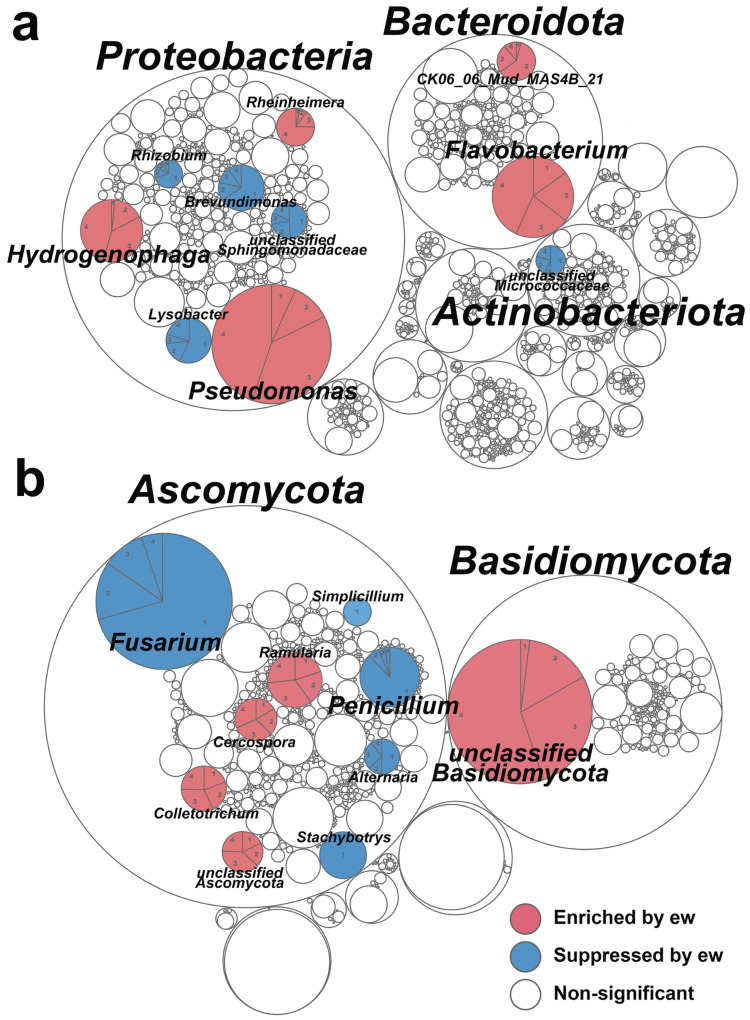
Substrate microbial communities’ compositions during the wheat cultivation process. (**a**) The differential relative abundance of bacterial communities. (**b**) The differential relative abundance of fungal communities. The outmost circles represent phylum level, and the inner circles represent genus; “1”, “2”, “3”, and “4” in each pie chart represent LS, LS+15ew, LS+30ew, and LS+45ew groups, respectively; the top three differential genus are considered as dominant genus and are highlighted in bold. The size of each bubble represents the relative abundance of microbial genera. Abbreviations: ew, earthworm(s).

To investigate the improvement mechanism from the perspective of the substrate’s microbial communities, 20 rhizosphere-adjacent substrate samples (4 types of cultivation substrate × 5 biological replicates) were subjected to amplicon sequencing; an average of 62,502 non-chimeric amplicon sequences were generated for the bacterial 16S rRNA gene and 69,970 for fungal ITS region, which were clustered into 27,054 bacterial ASVs and 9378 fungal ASVs. Through annotation, bacteria were classified into 3133 species, and fungi were classified into 1158 species. We analyzed the community composition at the phylum and genus levels. The dominant bacterial phyla were *Proteobacteria* and *Bacteroidota* (Figure 2a), while the dominant fungal phyla were *Ascomycota* and *Basidiomycota* (Figure 2b).

The linear discriminant analysis effect size (LEfSe) analysis (original results shown in Figure S2) identified the genera with significant differences between the pure lunar soil group and the earthworm group (Figure 2a). The top three abundant bacterial genera significantly enriched by the earthworms were *Pseudomonas*, *Flavobacterium*, and *Hydrogenophaga* (Figure 2a), which are generally reported as plant probiotic bacteria. Among these, *Pseudomonas* and *Hydrogenophaga* are known as phosphate-solubilizing bacteria, potentially converting phosphate into a plant-available form to support wheat growth. For top-abundant fungi (Figure 2b), the addition of earthworms significantly enriched the relative abundance of an unclassified *Basidiomycota* (NCBI: txid175245, 97% match) (rarely observed in the pure lunar soil simulant group), which is reported as a potential arbuscular mycorrhizal fungus (AMF), and significantly suppressed *Fusarium* and *Penicillium* (rarely observed in the earthworm group), which are generally reported as plant pathogens.

**Figure 3:**
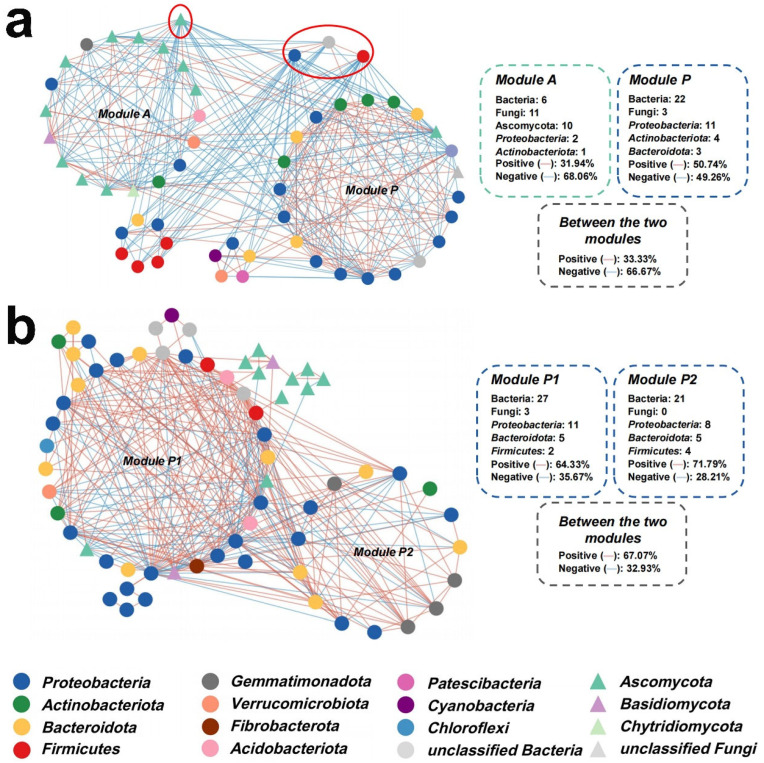
Substrate microbial communities’ interactions during the wheat cultivation process. (**a**) The molecular ecological network of the lunar soil simulant (earthworm-free, negative control) group. (**b**) The network of the earthworm-added groups. The circular nodes represent bacterial species, while the triangle nodes represent fungal ones; the nodes’ colors distinguish the phylum-level taxonomy; connectors are marked with red circles.

To investigate functional interactions within substrate microbial communities, we constructed two molecular ecological networks (pMENs) by integrating bacterial and fungal data, corresponding to the pure lunar soil simulant and earthworm groups (Figure 3a,b). As anticipated, both networks exhibited topological properties of small world, scale-free and modularity, and were significantly different from randomly generated networks (Table S1), indicating a qualified representativeness for complex system’s networks.

Notably, the pure lunar soil’s network had a higher number of fungal nodes which even formed a module itself (Figure 3a), whereas the earthworm group network was dominated by bacterial nodes with few fungal ones (Figure 3b). The earthworm group’s microbial network consisted of two major bacterial modules dominated by *Proteobacteria* nodes (thus named as “Module P1/P2”, Figure 3b), with *Bacteroidota* as the major secondary component; here, the few fungal nodes mainly presented in peripheral fragmented modules, suggesting a generally excluded status of the plant–pathogenic fungi from the substrate’s core functional microbial community. In contrast, in the pure lunar soil network, there was only one major bacterial module dominated by *Proteobacteria* (thus named as “Module P”, Figure 3a), and the other major module was dominated by the fungal phyla *Ascomycota* (named as “Module A”, Figure 3a), indicating a more significant role of fungi in the functional community.

Regarding the internal positive/negative correlations within the modules, the pure lunar soil’s network possessed a lower ratio (e.g., 32% in the fungal “Module A”, Figure 3a) of positive internal correlations than the earthworm’s network (Figure 3b), indicating extensive competition or conflicts among the microbes. The earthworm group’s network, in contrast, possessed a much higher ratio of positive correlations (both within and between the two major modules), reaching 64% and 72% (Figure 3b); these results indicate mutualistic symbiosis within the functional community, likely driven by external resource input from wheat root exudates. Moreover, based on the networks’ topology (Figure S3), the pure lunar soil’s network had four connector nodes linking the two modules, characterized by extensive negative correlations, indicating concentrated conflicts (Figure 3a); in contrast, the earthworm group network had no connector among the modules (Figure 3b).

Adding earthworms did not change bacterial α-diversity (Shannon index, Figure S4a), but significantly changed fungal α-diversity (Figure S4b); it went up and then down along the earthworm abundance, depicting a picture of fungi brought by earthworms from the feeding zone and then suppressed by their extensive digestive process.

### 2.3. The Improved Wheat Growth by Earthworms

**Figure 4:**
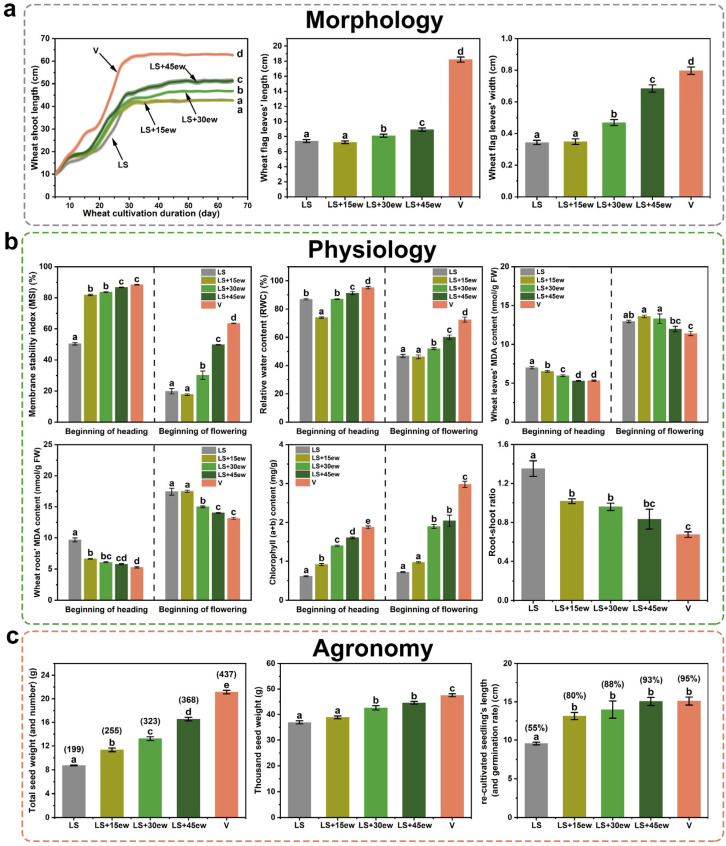
The wheats’ properties. (**a**) Wheat morphology, including the shoot lengths along the 65-day cultivation period, and the flag leaves’ lengths and widths at day 30. (**b**) Wheat physiology, where the heading stage indicates day 20, the flowering stage indicates day 40, and the root–shoot ratio was measured at the mature stage (day 65). (**c**) Wheat agronomy. Data are the means ± standard error, with *n* = 24 replicates in a, and *n* = 3 replicates in (**b**,**c**). Letters (i.e., a, b, c, ab, bc, cd, and d) close to the curves indicate the results of Wilcoxon rank–sum tests identifying significant differences (*p* < 0.05) among the labeled curves, and those above the bars indicate the results of ANOVA’s post hoc LSD tests (*p* < 0.05).

To assess the substrates’ cultivability with/without earthworms, the whole life span (“from seed to seed”) of wheat cultivation, initiated simultaneously with the addition of earthworms, was observed across morphological, physiological, and agronomic dimensions, showing a generally coordinative improvement through earthworm addition (Figure 4). Morphologically, wheat shoot length, flag leaf length, and flag leaf width were all significantly and successively improved by the earthworm abundance gradient, where, notably, the 45-earthworm-group wheat shoot length reached 82% of the vermiculate cultivated wheat (Figure 4a).

Physiologically, at the heading stage of wheat, all indicators showed improvements with the increased abundance of earthworms, including the Membrane Stability Index (MSI), the Relative Water Content (RWC), malondialdehyde (MDA) content, and chlorophyll content (Figure 4b). However, later, at the flowering stage, these indicators in the earthworm-added groups became less ideal than those at the heading stage (although still better than the pure lunar soil simulant) (Figure 4b), possibly caused by the earthworm population’s gradual death along the wheat cultivation. Additionally, at the harvest stage, the root–shoot ratio in the earthworm treatments (1.02, 0.96, and 0.83, respectively) was all significantly lower than that of the pure lunar soil simulant group (1.35), and the high-earthworm-abundance group (0.83) had no significant difference compared to the vermiculite group (0.67, as an ideal control substrate) (Figure 4b), which indicates the wheat’s resource allocation tendency to the stem, leaf, and seed development rather than overcoming the root’s water, nutrient, or oxygen deficiencies, thus suggesting a mitigated stress from the substrate’s salinization and compaction.

Agronomically, regarding the seeds produced by the cultivated wheat, the total seed weight, total seed number, and thousand seed weight all increased with the abundance of earthworms (Figure 4c), with the high-earthworm-abundance group’s indicators reaching 78%, 84%, and 94% of those in the vermiculite group (Figure 4c). Regarding the seeds’ reproduction, the pure lunar soil simulant group exhibited a germination rate of only 55%, with an average seedling length of 9.55 cm after 7 days of growth (Figure 4c), and seed blackening was even observed during the germination; in contrast, the seed germination rate of the low-earthworm-abundance group already increased to 80%, with an average seedling length of 13.14 cm on day 7 (Figure 4c), and the high-earthworm-abundance group (93%, 15.05 cm) even had no significant difference compared to the vermiculite-cultivated group (95%, 15.09 cm), indicating a high quality of seeds from the earthworm treatments (Figure 4c) and thus a high potential for sustainable lunar agriculture.

### 2.4. Improvement Mechanisms Explained by Ecological Models

**Figure 5:**
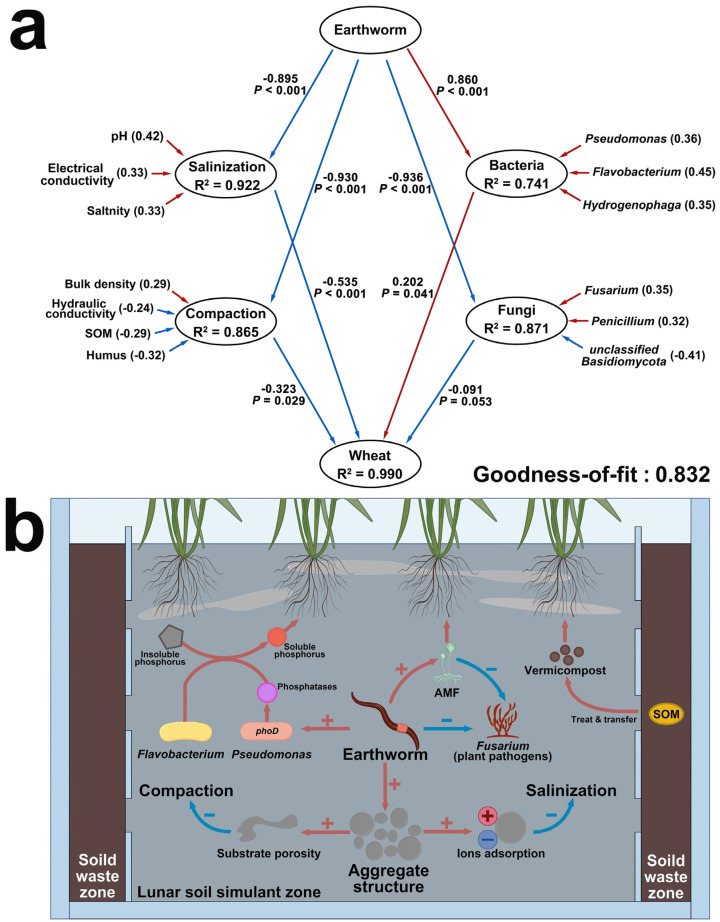
The Partial Least Squares Path Model (PLS-PM) (**a**) and the hypothesized improvement mechanism (**b**) of the total story.

To mathematically describe the total relationship among the four parties (substrate, microbes, wheat, and earthworms), and to explore the potential mechanisms of the improvement, we conducted several ecological models, where six most abundant and notably differential microbial genera (Figure 2a,b) were manually chosen for the models (Figure 5).

To briefly outline the relationships, a redundancy analysis (RDA) and Mantel test were performed first (Figure S5). The RDA suggested a larger impact from the compaction (bulk density, humus, and SOM, ~RDA1) than the salinization (electrical conductivity and salinity, ~RDA2) onto the microbial communities, where adding only low-abundance earthworms (the 15 ew group) significantly induced the salinization (Figure S5a). Taking the wheat into consideration, the Mantel test found that salinization significantly inhibited the wheat’s growth in its early and middle stages (~morphological properties), while *Flavobacteria* and the AMF (unclassified *Basidiomycota*) are able to mitigate this inhibition (Figure S5b). Compaction (bulk density and reduced humus) and fungal diseases (*Fusarium* and *Penicillium*) significantly inhibited wheat in its middle stage (~physiological properties) and late stage (~agronomic properties, Figure S5b). It is worth noting that the AMF had a promoting effect throughout the entire wheat life cycle (on all three properties), suggesting the strong colonization ability of AMF into the roots.

Guided by these findings, a Partial Least Squares Path Model (PLS-PM) was performed among the four parties to describe the overall mechanisms. The model’s goodness-of-fit of 0.832 indicates a good fit (>0.7) (Figure 5a). According to the model, earthworms significantly inhibited salinization and compaction (*p* < 0.001) and altered the composition of the substrate microbial community (*p* < 0.001). Based on this, all four latent variables have a significant effect on the wheat, with the most significant inhibition being due to salinization (*p* < 0.001, weight = −0.535), followed by compaction (*p* = 0.029, weight = −0.323); the promotional/inhibitory effects of the bacterial/fungal community on wheat are weaker (weight = 0.202 and −0.091, respectively), indicating the earthworms’ greater improvement on the substrate’s salinization and compaction, rather than through the microbial community. The hypothesized total mechanism of this study is thus visualized in Figure 5b.

## 3. Discussion

In lunar-based BLSSs, the primary materials available for plant cultivation substrates are lunar soil and organic solid waste [9]. Lunar soil (and its simulants) can act as an inert dilution agent to mitigate salinization. However, due to its fine-grained, high-density nature and lack of organic matter or aggregate structures, it is prone to hardening and compaction. This compaction disrupts soil aggregates through surface drying and crusting after irrigation [14]. On the other hand, organic solid waste, which is highly lignocellulosic, can serve as an organic amendment to form aggregate structures. However, in a BLSS, which requires rapid and efficient material cycling to meet life-support demands, solid waste is often incompletely decomposed. Additionally, the high salinity from plant nutrient solutions can induce root ion stress and promote crop pathogens [9]. The RDA illustrates that compaction and salinization originate from lunar soil simulant and solid waste, respectively. Compaction indicators are associated with the lunar soil simulant group, while salinization indicators are linked to the 15-earthworm group (which exhibited the highest salinity, likely due to the solid waste introduced by earthworms) (Figure 1b,c). These two factors are distinctly separated, as indicated by the considerable angle between them in the RDA plot (Figure S5). Although earthworms are typically sensitive to high-salinity substrates and ionic stress [15], the buffering effect of lunar soil simulant likely promote their survival, and the formed aggregates (Figure S1) further improve ion adsorption (reducing salinization) and increase substrate porosity (mitigating compaction) (Figure 5b). Soil acidity typically destabilizes aggregates, but the alkaline nature of lunar soil (Figure 1a) may actually benefit aggregate stability [16]. Additionally, low soil carbon content often reduces the functional stability of soil aggregates [17]. The soil organic matter (SOM) and humus introduced by earthworms from the solid waste zone (Figure 1g,h) likely sustained the structure of the aggregates (e.g., shape, size, and binding energy between organomineral particles) [18]. Thus, a complementary improvement mechanism emerges, where lunar soil simulant and solid waste address compaction and salinization, respectively, with earthworms catalyzing the formation of soil aggregates.

From a microbiological perspective, the microbial phylogenetic molecular ecological networks (pMENs) in the presence of earthworms exhibited reduced fungal involvement and increased positive correlations (Figure 3a,b). Interestingly, the microbial network of pure lunar soil (Figure 3a) resembles rhizospheric networks around infected plant roots, characterized by a high proportion of fungal nodes (predominantly *Ascomycota*) and numerous negative correlations, indicating an antagonistic pattern typical of unhealthy root systems [19]. In contrast, the earthworm-amended group’s microbial network resembles that of healthy plant rhizospheres, with bacteria dominating the network. This suggests that wheat recruits rhizospheric symbiotic bacteria to compete with fungi [20]. Molecular ecological networks reflect functional interactions among microbes, with modules representing independent functional units [21]. From a game theory perspective, the abundance of positive correlations in the earthworm-amended group (Figure 3b) aligns with an incremental (positive-sum) pattern, likely driven by continuous root exudate supply as a microbial nutrient source [22]. In contrast, the numerous negative correlations in pure lunar soil (Figure 3a) suggest a stock (zero-sum) pattern, indicative of poor root exudation. Combined with the high fungal participation, this pattern may result from rotten roots (caused by severe compaction) serving as an organic nutrient source for fungi [23].

The rotten roots hypothesis is further supported by fungal dynamics (Figure 2b). Specifically, earthworms significantly suppressed *Fusarium* and Penicillium while enriching unclassified *Basidiomycota* (Figure 2b). *Fusarium* is a harmful plant pathogen that causes head blight, root rot, and wilt by secreting cell-wall-degrading enzymes and toxins that disrupt plant cell function [24]. *Penicillium*, often recruited by plants under stress, can produce plant hormones, improve antioxidant enzyme activity, and improve nutrient absorption [25], explaining its enrichment in the stressful pure lunar soil simulant (Figure 2b). The unclassified *Basidiomycota*, identified via 16S rRNA gene sequencing, is likely an arbuscular mycorrhizal fungus (AMF) [26]. AMFs form symbiotic associations with plants, improving phosphorus absorption, salt tolerance, and pathogen inhibition, making them valuable for saline soil bioremediation [27]. The enrichment of plant–probiotic AMFs by earthworms is a promising development for lunar soil cultivation.

For bacteria, earthworms significantly enriched three genera: *Pseudomonas*, *Flavobacterium*, and *Hydrogenophaga* (Figure 2a). All of these are known plant-growth-promoting bacteria. *Pseudomonas* inhibits plant pathogens, synthesizes growth hormones, improves disease resistance, and forms mutualistic symbioses with plants [28]. Additionally, lunar soil is rich in mineral phosphorus of plant-unavailable forms [29], but *Pseudomonas*, as a phosphate-solubilizing bacterium, can convert these into plant-available forms [30] (Figure 5b). *Flavobacterium* suppresses fungal diseases by producing antifungal enzymes and plant resistance inducers [31]. *Hydrogenophaga* promotes plant growth by producing growth factors, solubilizing phosphates, removing heavy metals, and enhancing nitrogen-use efficiency [32]. In summary, earthworms suppressed pathogens and enriched plant probiotics (Figure 2a,b), likely contributing to improved wheat performance (Figure 5a).

The combined improvements in substrate properties and microbial communities ultimately improved wheat cultivation. Under stress, wheat typically reduces height and flag leaf area to minimize transpiration and respiration [33]. Consistent with this, wheat in the pure lunar soil group exhibited poor morphology (Figure 4a). However, earthworms mitigated salinization and compaction (Figure 1), improving wheat growth (Figure 4a), as evidenced by the improved membrane stability, water retention, antioxidant capacity, photosynthesis, and root–shoot ratio. Specifically, shoot length reached 82% of that in the vermiculite group (Figure 4a), a significant improvement over our previous trial without earthworms (64%) [9], demonstrating the efficacy of earthworms. Importantly, seed quality approached that of the vermiculite-cultivated group (Figure 4c), indicating strong generational transmission efficiency and long-term potential for lunar agriculture. However, the reduced prominence of earthworm-added groups around the flowering stage (Figure 4b) raises concerns about earthworm mortality during cultivation and the need for future remedial measures.

The heterogeneous-substrate design, with solid waste placed diagonally across the cultivation area (Figure 5b), provided earthworms with diverse habitats and food sources, enhancing their survival and activity [34,35]. This design aimed to maximize earthworm burrowing and feeding behaviors, promoting aggregate formation and gradual mixing of lunar soil simulant and solid waste. This approach buffered potential stresses on wheat, mitigating both compaction and salinization. We anticipate that this study will inspire similar soil remediation efforts on Earth, particularly for salinized, compacted, or desertified soils.

## 4. Materials and Methods

### 4.1. Lunar Soil Simulant

The lunar soil simulant utilized in this study was CUG-1B, which was developed by Prof. Long XIAO’s team, China University of Geosciences (Wuhan). The physical properties and chemical composition of CUG-1B closely resemble those of real lunar soil samples collected during the Apollo and CE-5 missions (Table 1), indicating its suitability for the research objectives of this study [36].

### 4.2. Earthworms and Their Feeding

*Eisenia fetida*, a commonly used laboratory model species, was chosen for improving the lunar soil cultivation substrates, leveraging its robust adaptability and rapid reproductive rate [12]. Earthworms were procured from a commercial earthworm culture farm (Changzhou, Jiangsu, China), and acclimatized for a two-week period in lunar soil simulant while being fed with organic solid waste in the laboratory prior to the commencement of the experiment (Figure S6). This acclimatization process ensured the earthworms’ adaptation to the next-step cultivation experiments. In the experiment, fully developed adult earthworms (0.50 ± 0.039 g, consistent with the typical size range of *Eisenia fetida* [37]) with clitellum were utilized following a thorough sanitization process to ensure their effectiveness in improving soil properties and to prevent the introduction of pathogens and pests into the cultivation substrate.

Earthworms fed on the organic solid waste from the “Lunar Palace 365” experiment, the world’s longest, closed, stable-crewed BLSS experiment [38]. The composition of solid waste included aerobically fermented plant residues (~85% *w*/*w*), human feces (~15% *w*/*w*), and their accompanying microbes, reflecting the waste composition expected in crewed lunar bases. Due to its high moisture content, clumpy texture, and salinization, the original solid waste was considered unsuitable for earthworms’ habitation. To address this issue, the original solid waste was desiccated at 30 °C for 48 h to achieve a moisture content of ≤5% (to accelerate the decomposition and maturation) [9], then pulverized and homogenized with lunar soil simulant. The final mixing ratio of the processed solid waste to lunar soil was adjusted to 1:4 (*w*/*w*) (determined as the optimal ratio for earthworm feeding through preliminary experiments). During the cultivation period, earthworms would gradually transform the mixture into vermicompost through their feeding and excretion activities, and the vermicompost was then mixed with the substrate as well.

### 4.3. Wheat Cultivation Experiment

The wheat used in this study was spring wheat (*Triticum aestivum* L.). Wheat seeds were sterilized by soaking in 0.1% KMnO_4_ solution for 15 min, and then placed in Petri dishes containing a small amount of sterile water for 36 h at 20–25 °C for germination. Five experimental groups of wheat cultivation substrates were applied in total: vermiculite (V) as the optimal growth substrate (positive control), pure lunar soil simulant without earthworms (LS, negative control), and lunar soil simulant introducing 15 (LS+15ew), 30 (LS+30ew), and 45 (LS+45ew) earthworms (Figure S7). Each experimental group had 4 biological replicates, with 60 seedlings per replicate (Table S2). Germinated seeds were transplanted into substrates and cultivated under 24 ± 1.5 °C and a 24 h photon flux of 600 μmol/(s∙m^2^), which simulate the optimal light condition for wheat growth in BLSS [39]. In the two types of earthworm-free cultivation substrates (V and LS), a daily irrigation of 200 mL Hoagland nutrient solution was applied to provide essential macro- and micro-nutrients (e.g., N, P, K, Ca, Mg, S, Fe, Mn, Zn, Cu, B, and Mo) for wheat growth [40]. This solution compensates for the lack of nutrients in V and LS. In the earthworm-added substrates (LS+15ew, LS+30ew, LS+45ew), 200 mL of distilled water was applied daily, as the organic waste mineralization mediated by earthworms should have supplied sufficient nutrients.

Cultivation containers are acrylic boxes (25 × 15 × 15 cm, with 20 drainage holes with a diameter about 2 mm at the bottom). Given that the direct contact between solid waste (with high levels of salinity) and wheat is likely to result in adverse effects, two supplementary compartments (5 × 5 × 15 cm) were separated at two diagonal corners of the cultivation container by a right-angle baffle (with 12 holes with a diameter about 1 cm for earthworms passage), regarded as feeding zones in the three earthworm treatment groups. The mixture of solid waste and lunar soil simulant was put into the feeding zones.

### 4.4. Wheat Parameters Measurement

To assess the morphology of wheat, shoot lengths were measured every two days starting from the fifth day of cultivation using a straight scale. On the 30th day, when all wheat plants were in the heading stage, the length and width of flag leaves were measured using a straight scale. Twelve samples of wheat plants were randomly selected for measurement.

Physiologically, leaves’ MSI, leaves’ RWC, leaves and roots’ MDA content, and leaves’ chlorophyll content of wheat were separately measured at the beginning of heading and flowering stage; the root–shoot ratio was measured at the harvest stage. The MSI of leaves was determined by quantifying electrical conductivity of wheat leaf leachates immersed in double-distilled water at temperatures of 40 °C and 100 °C [41]. The RWC was determined by measuring wheat leaf samples’ fresh weight, turgid weight, and dry weight [42]. The leaves’ and roots’ MDA content of wheat was determined using a MDA Content Test Kit (A003-1-1, Nanjing Jiancheng, China). The chlorophyll content of leaves was determined by ultraviolet spectrophotometer (SP-752, Shanghai spectrum instruments Co., Ltd., Shanghai, China) [43]. At the harvest stage, fresh samples were dried in an oven at 75 °C until a constant weight was achieved, and the dry weight of the roots and shoots were separately measured using an analytical balance [44].

To assess the production and seed quality (agronomy) of wheat, artificial threshing was performed at day 65 which corresponds to wheat maturity, and the total seed weight and thousand seed weight were measured using an analytical balance for wheat subjected to five distinct treatments [45]. Subsequently, the harvested seeds were soaked, germinated, and re-cultivated, with the germination rate and shoot length on the 7th day for wheat under the five treatments to test the reproduction.

### 4.5. Substrates’ Physical/Chemical Properties

All lunar soil simulant samples (25 ± 2.5 g) for analysis were extracted by a non-disturbed soil sampler. The pH, electrical conductivity and salinity were determined by preparing substrates’ leachates, followed by measurement using a pH meter (FE28, METTLER TOLEDO, Greifensee, Switzerland), an electrical conductivity meter (FE38, METTLER TOLEDO, Greifensee, Switzerland), and a salinity meter (AZ8371, AZ Instrument, Taiwan, China). The bulk density was determined using Archimedes’ method in the graduated 50 mL cylinder [46]. The SOM was determined using the potassium dichromate volumetric [47]. The soil humus content was determined by potassium chromate oxidation and external heating method after extraction with 0.1 mol/L sodium pyrophosphate and 0.1 mol/L NaOH. The total carbon and nitrogen were determined using an EA element analyzer (UNICUBE, Elementar, Langenselbold, Germany). The hydraulic conductivity was determined by the constant head method and Henri Darcy’s law [48].

### 4.6. Sequencing Analysis of the Substrates’ Microbial Community

Comprehensive information about the microbial community composition in cultivation substrate was obtained through 16S rRNA high-throughput sequencing. DNA extraction, PCR amplification, and sequencing of cultivation substrate microbial communities were performed by Biomarker Tech. Corp., Beijing, China. Briefly, DNA was extracted with the TGuide S96 Magnetic Soil/Stool DNA Kit (Tiangen Biotech [Beijing] Co., Ltd., Beijing, China) according to the manufacturer’s instruction. DNA concentration was measured with the Qubit dsDNA HS Assay Kit and Qubit 4.0 Fluorometer (Invitrogen, Thermo Fisher Scientific, Eugene, ON, USA). The 338F: 5′-ACTCCTACGGGAGGCAGCA-3′ and 806R: 5′-GGACTACHVGGGTWTCTAAT-3′ universal primer set was used to amplify the V3-V4 region of 16S rRNA gene. Both the forward and reverse primers were tailed with sample-specific Illumina index sequences. The PCR was performed in a total reaction volume of 10 μL: DNA template 5–50 ng, *Vn F (10 μM) 0.3 μL, *Vn R (10 μM) 0.3 μL, KOD FX Neo Buffer 5 μL, dNTP (2 mM each) 2 μL, KOD FX Neo 0.2 μL, and ddH_2_O up to 10 μL. Vn F and Vn R were selected according to the amplification area. The initial denaturation at 95 °C for 5 min was followed by 25 cycles of denaturation at 95 °C for 30 s, annealing at 50 °C for 30 s and extension at 72 °C for 40 s, and a final step at 72 °C for 7 min. PCR amplicons were purified with Agencourt AMPure XP Beads (Beckman Coulter, Indianapolis, IN, USA) and quantified using the Qubit dsDNA HS Assay Kit and Qubit 4.0 Fluorometer (Invitrogen, Thermo Fisher Scientific). The quantified amplicons were then pooled together in equal amounts for the library. The constructed library was sequenced using Illumina NovaSeq 6000 (Illumina, Santiago, CA, USA).

Downstream sequencing analysis was performed on BMK Cloud (Biomarker Technologies Co., Ltd., Beijing, China). Raw sequences were first processed using Trimmomatic and FLASH, with a moving window of 50 bp and a quality threshold score of 30. Singletons were then removed. Next, high-resolution amplicon sequence variants (ASVs) were identified from the reads using DADA2 (version 1.34) [49]. Lastly, a representative sequence of each ASV was annotated through SILVA ribosomal RNA gene database (version 138.2) with a confidence score of 0.7 [50].

### 4.7. Molecular Ecological Network Analysis

With 16S rRNA gene (bacteria) ASVs, ITS ASVs and environmental factors pooled together as the input, phylogenetic molecular ecological networks (pMENs) were constructed based on random matrix theory (RMT)-based network [21]. The threshold of similarity coefficients (r values of the Spearman’s rho correlation) for network construction was automatically determined when the nearest-neighbor spacing distribution of eigenvalues transitioned from Gaussian orthogonal ensemble to Poisson distributions [21]. Random networks corresponding to all pMENs were constructed using the Maslov–Sneppen procedure with the same network size and average link number to verify the system-specificity, sensitivity, and robustness of the empirical networks [51]. Network graphs were visualized with Cytoscape 3.8 software.

### 4.8. Statistical Analyses

Shannon index (α-diversity) was calculated and displayed by QIIME2 and package “ieggr” (version 4.17) in R software (version 4.3.3). Analysis of variance (ANOVA) was conducted using GraphPad Prism 9.5 software and its post hoc Fisher’s Least Significant Difference (LSD) test was conducted using the package “vegan” (version 2.6-8) in R software; these were used to examine the significant differences among seed or plant weights. Redundancy analysis (RDA) was performed by package “vegan” in R software to detect relationships among microbial communities, environmental factors, and wheat growth parameters. The linear relationship between two variables was evaluated by calculating the Spearman correlation coefficient, and the linear relationship between two matrices was tested using the Mantel test based on the Bray–Curtis algorithm by package “vegan” in R software [52]. The PLS-PM were constructed by “plspm” function in R to investigate relationships among relative abundance of key microbes, substrates’ physical/chemical properties, and wheat parameters [53].

## 5. Conclusions and Future Directions

Lunar soil lacks both nutrients and aggregate structures, leading to compaction. While organic solid waste can mitigate compaction and provide nutrients, its salinization poses a threat, which lunar soil can buffer. We propose a conceptual framework for lunar soil improvement, emphasizing its complementary relationship with organic waste and introducing earthworms as a novel solution. Earthworms enriched soil aggregates, improving porosity and ion adsorption capacity, and significantly mitigated compaction and salinization (Figure 1). The microbial community shifted toward a cooperative mode dominated by plant probiotics, including an arbuscular mycorrhizal fungus (Figure 2b). These together improved the cultivated wheat’s morphology, physiology, and agronomy (including the production), reaching ~80% of the vermiculite + nutrient solution group (Figure 4) with a concise mechanism depiction (Figure 5); this is much better than the ~64% performance of our previous without-earthworm trial [9]. This study lays practical groundwork for future lunar agriculture while offering fresh perspectives on the dynamic interactions between plants and microbes in such environments. Future research should focus on optimizing the earthworm-mediated soil improvement process under simulated lunar conditions, including varying gravity, radiation, and magnetic field levels, to better understand their adaptability and long-term efficacy.

## Figures and Tables

**Table 1:** Physical properties and chemical composition (% *w*/*w*) of the lunar soil simulant CUG-1B and two real lunar soil samples.

Property	CUG-1B	Apollo Lunar Soils	CE-5 Lunar Soil
Physical properties			
Equivalent diameter (μm)	75	24–429	4.84–432.27
Median grain size (μm)	60	28–493	52.54–55.24
Bulk density (g/cm^3^)	1.59	0.87–1.93	1.24
True density (g/cm^3^)	2.71	2.9–3.44	3.20
Cohesion (kPa)	0.14–1.59	0.26–1.8	No data available
Internal friction angle (°)	33.6–35.5	25–50	No data available
Void ratio	0.52–0.95	0.67–2.37	No data available
Chemical composition (% *w*/*w*)			
SiO_2_	48.11	42.2–48.1	42.2
TiO_2_	2.64	0.53–7.8	5.00
Al_2_O_3_	14.15	13.6–27.3	10.8
FeO_T_	12.45	5.1–15.3	22.5
MnO	0.19	0.14–0.3	0.28
MgO	5.81	5.7–9.4	6.48
CaO	7.42	10.7–15.7	11.0
Na_2_O	4.67	0.46–0.7	0.26
K_2_O	2.55	0.16–0.55	0.19
P_2_O_5_	0.92	0.05–0.51	0.23
Total	98.92	99.48–99.6	98.94

## Data Availability

The original data presented in the study are openly available in Zenodo, a publicly accessible repository at https://doi.org/10.5281/zenodo.14886256 (accessed on 18 February 2025). The script used for analysis and visualizations of the data used for this study and raw data are available from the authors upon reasonable request.

## Supplementary Materials

The following supporting information can be downloaded at: https://www.mdpi.com/article/10.3390/plants14071046/s1, Table S1. Major topological properties of the empirical networks (pMENs) of the earthworm-free (lunar soil simulant) and earthworm-added groups and their corresponding random networks. Table S2. Experimental design for wheat cultivation in different substrates. Figure S1. The side view of the earthworm-added cultivation substrate on day 5 and day 50, with the aggregate structure produced by earthworms marked with the red arrow. Figure S2. LEfSe analysis which distinguishes the substrate microbial community’s relative-abundance-differential genus among different treatments (genus level). (a) bacterial LEfSe (LDA = 3.5). (b) fungi LEfSe (LDA = 3.5). Figure S3. The distribution of network nodes based on their topology. The symbols represent nodes in earthworm group (red dots) and no-earthworm group (blue dots) networks. The threshold values of *Z_i_* and *P_i_* for categorizing nodes are 2.5 and 0.62, respectively. *Z_i_* > 2.5 and *P_i_* > 0.62 indicates network hubs; *Z_i_* > 2.5 and *P_i_* ≤ 0.62 indicate module hubs; *Z_i_* ≤ 2.5 and *P_i_* > 0.62 indicate connectors; and *Z_i_* ≤ 2.5 and *P_i_* ≤ 0.62 indicate peripherals. Figure S4. Microbial alpha diversity analysis expressed as Shannon index. (a) Bacterial Shannon index. (b) Fungal Shannon index. Figure S5. Redundancy Analysis (RDA) and Mantel test sketching the relationships among the major parties. (a,b) RDA of physical/chemical factors and microbial communities, including bacterial (a) and fungal (b). (c) Two-sided Mantel test among key genus, physical/chemical factors and wheat properties. Figure S6. Eisenia fetida earthworms after acclimatization, ready for introduction into the cultivation substrates. Figure S7. Photographic documentation of the experimental setup for each treatment in wheat cultivation.
