# Supplementary material for: Improving Lunar Soil Simulant for Plant Cultivation: Earthworm-Mediated Organic Waste Integration and Plant-Microbe Interactions
Source: Plants (Basel). 2025 Mar 27;14(7):1046. doi: 10.3390/plants14071046 (PMC11990861; doi:10.3390/plants14071046)
Supplement: Supplementary file 1 [file plants-14-01046-s001.zip › plants-3508934-supplementary.pdf]

Table S1. Major topological properties of the empirical networks (pMENs) of the earthworm-free (lunar soil simulant) and earthworm-added groups and their corresponding random networks.

Network name	Empirical networks								Random networks[a]		
	No. of original ASVs	Similarity threshold	Network size	R^2 of power law	Average connectivity	Average path distance (GD)	Average clustering coefficient	Modularity (No. of modules)	Average path distance ± SD	Average clustering coefficient ± SD	Average modularity± SD
ew-free	13,256	0.74	55	0.380	11.382	2.003[b]	0.301[b]	0.148[b]	1.879+/-0.045	0.331+/-0.019	0.089+/-0.008
ew-added	29,087	0.73	72	0.479	9.736	2.428[b]	0.313[b]	0.162[b]	2.163+/-0.030	0.357+/-0.023	0.111+/-0.017

[a]The random networks were generated by rewiring all the links of a network with an identical number of nodes and links to the corresponding empirical network.

[b]Significant difference ($p < 0.050$) between empirical networks and random networks.

Table S2. Experimental design for wheat cultivation in different substrates.

Experimental group	Abbreviation	Number of biological replicates	Number of wheat seedlings
Vermiculite	V	4	60
Lunar soil simulant	LS	4	60
Lunar soil simulant + 15 earthworms	LS+15ew	4	60
Lunar soil simulant + 30 earthworms	LS+30ew	4	60
Lunar soil simulant + 30 earthworms	LS+45ew	4	60

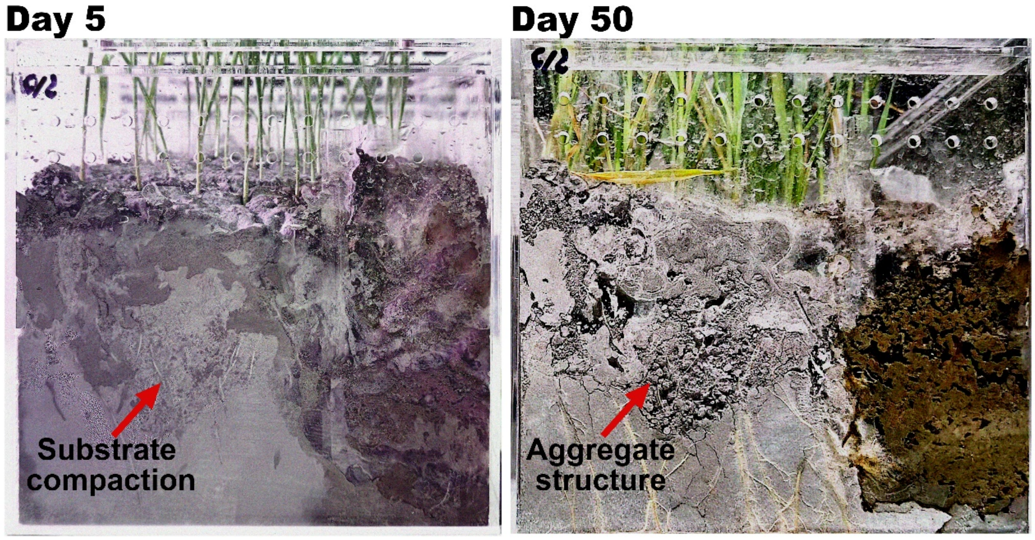

Figure S1. The side view of the earthworm-added cultivation substrate on day 5 and day 50, with the aggregate structure produced by earthworms marked with the red arrow.

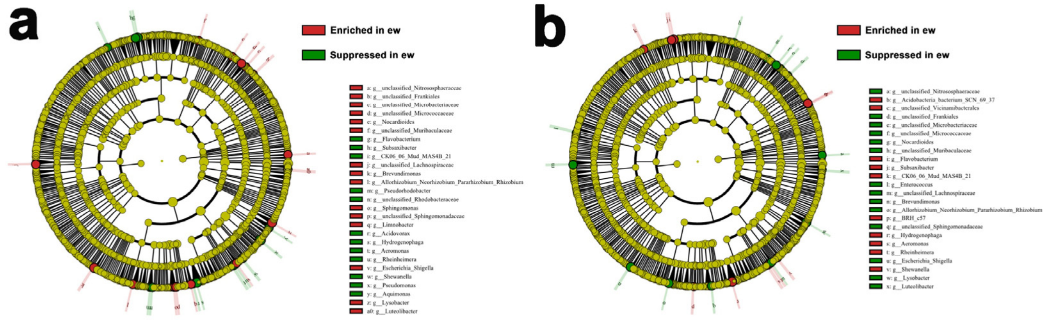

Figure S2. LEfSe analysis which distinguishes the substrate microbial community's relative-abundance-differential genus among different treatments (genus level). a, bacterial LEfSe (LDA = 3.5). b, fungi LEfSe (LDA = 3.5).

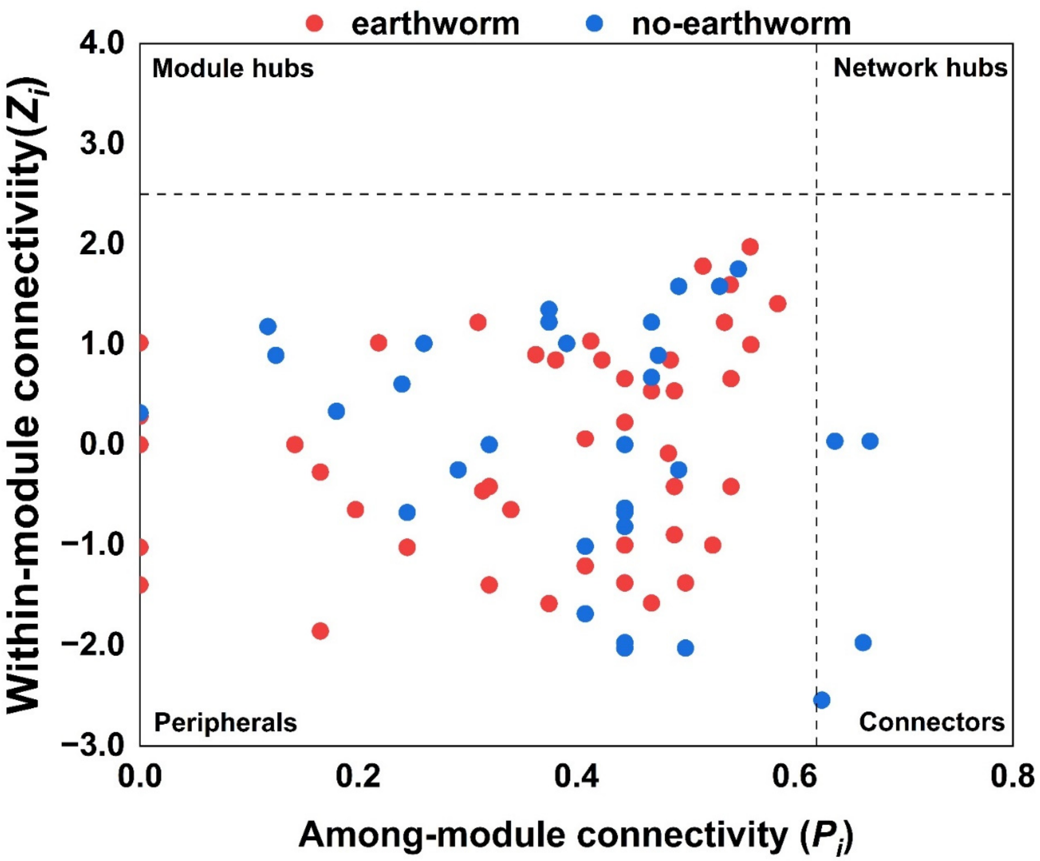

Figure S3. The distribution of network nodes based on their topology. The symbols represent nodes in earthworm group (red dots) and no-earthworm group (blue dots) networks. The threshold values of Z_i and P_i for categorizing nodes are 2.5 and 0.62, respectively. $Z_i > 2.5$ and $P_i > 0.62$ indicates network hubs; $Z_i > 2.5$ and $P_i \leq 0.62$ indicate module hubs; $Z_i \leq 2.5$ and $P_i > 0.62$ indicate connectors; and $Z_i \leq 2.5$ and $P_i \leq 0.62$ indicate peripherals.

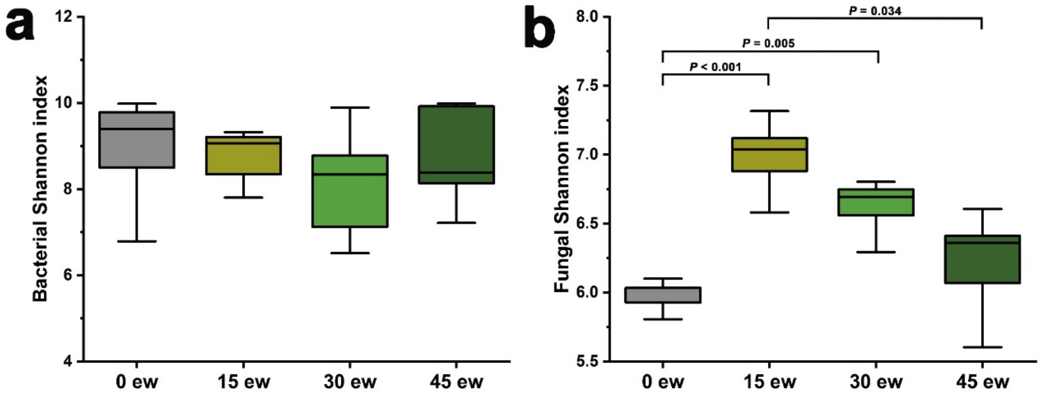

Figure S4. Microbial alpha diversity analysis expressed as Shannon index. a, Bacterial Shannon index. b, Fungal Shannon index.

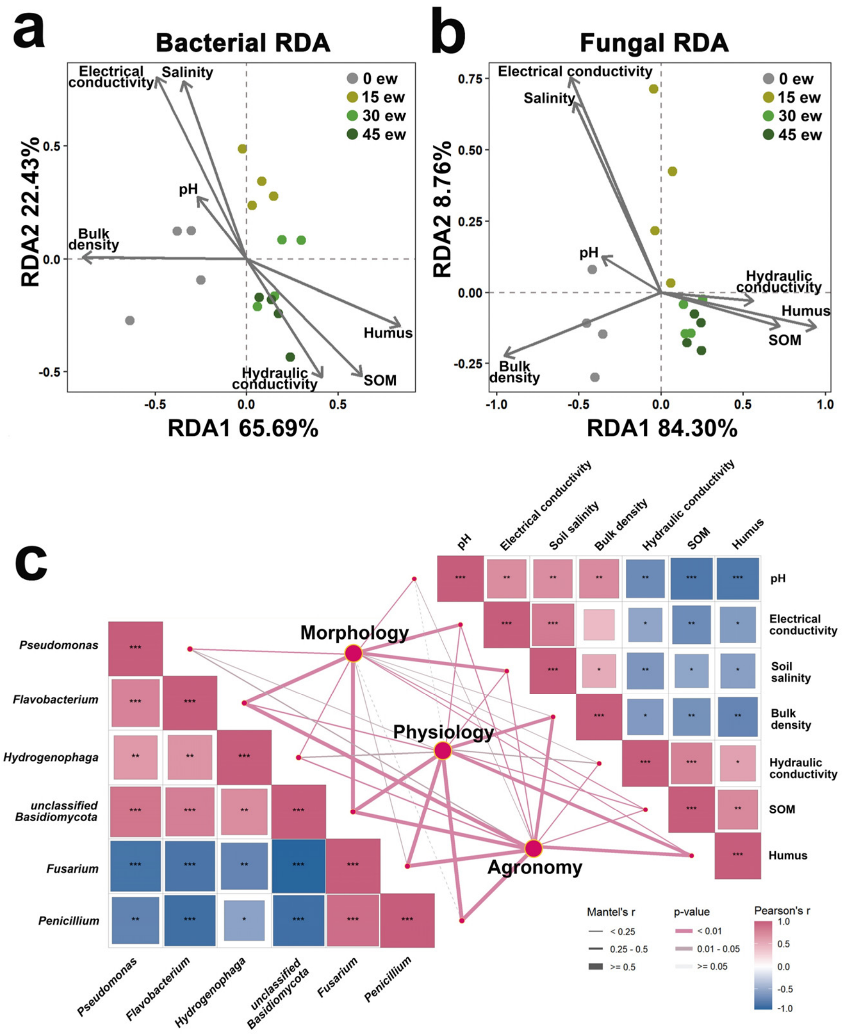

Figure S5. Redundancy Analysis (RDA) and Mantel test sketching the relationships among the major parties. a,b, RDA of physical/chemical factors and microbial communities, including bacterial (a) and fungal (b). c, Two-sided Mantel test among key genus, physical/chemical factors and wheat properties.

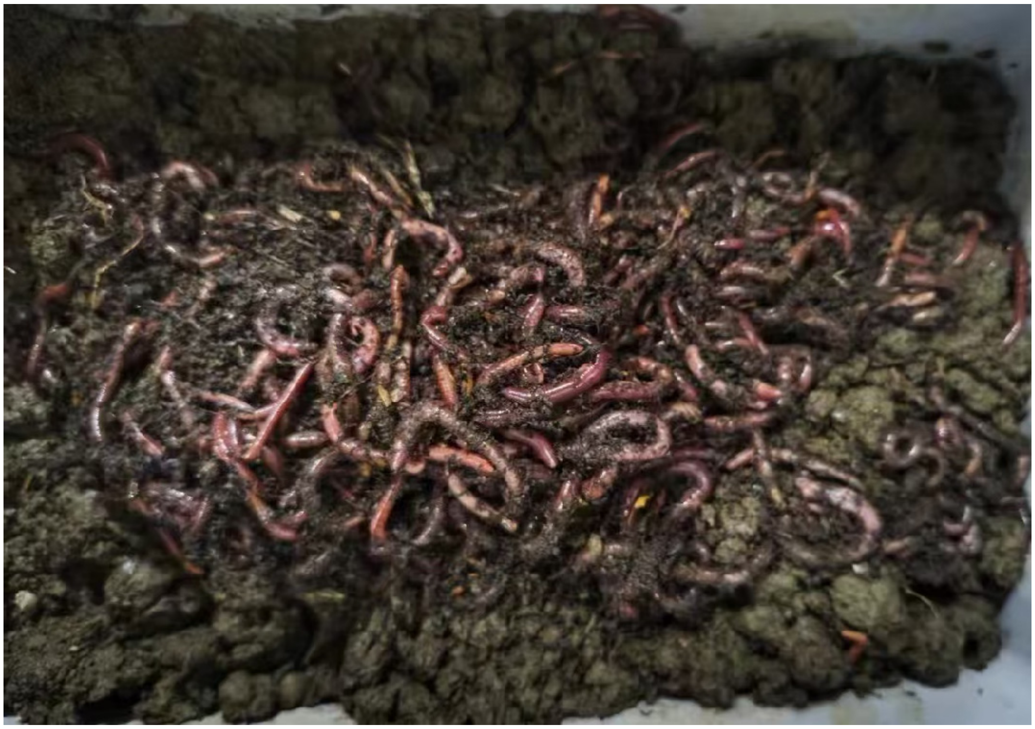

Figure S6. Eisenia fetida earthworms after acclimatization, ready for introduction into the cultivation substrates.

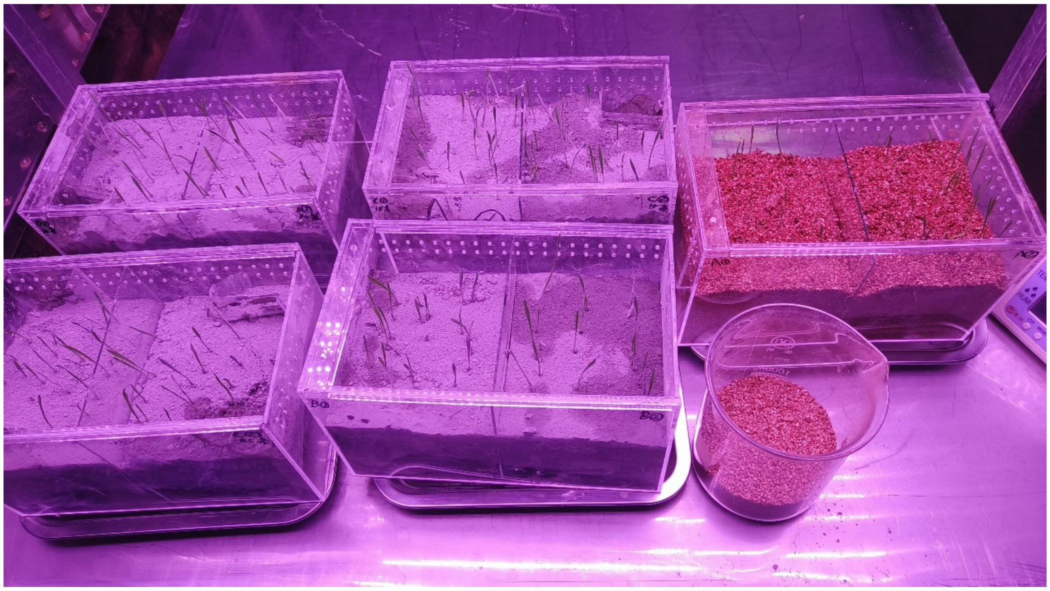

Figure S7. Photographic documentation of the experimental setup for each treatment in wheat cultivation.
